# An Introduction to Zeolite Synthesis Using Imidazolium-Based Cations as Organic Structure-Directing Agents

**DOI:** 10.3390/molecules22081307

**Published:** 2017-08-06

**Authors:** Paloma Vinaches, Katia Bernardo-Gusmão, Sibele B. C. Pergher

**Affiliations:** 1 LABPEMOL, Institute of Chemistry, Universidade Federal do Rio Grande do Norte (UFRN), Av. Senador Salgado Filho, 3000, Lagoa Nova, 59078-970 Natal/RN, Brazil; palomavinaches@gmail.com; 2 LRC, Institute of Chemistry, Universidade Federal do Rio Grande do Sul (UFRGS), Av. Bento Gonçalves, 9500. P.O. BOX 15003, 91501-970 Porto Alegre/RS, Brazil; katiabg@iq.ufrgs.br

**Keywords:** imidazolium-based cations, zeolite synthesis, structure-directing agents

## Abstract

Zeolite synthesis is a wide area of study with increasing popularity. Several general reviews have already been published, but they did not summarize the study of imidazolium species in zeolite synthesis. Imidazolium derivatives are promising compounds in the search for new zeolites and can be used to help understand the structure-directing role. Nearly 50 different imidazolium cations have already been used, resulting in a variety of zeolitic types, but there are still many derivatives to be studied. In this context, the purpose of this short review is to help researchers starting in this area by summarizing the most important concepts related to imidazolium-based zeolite studies and by presenting a table of recent imidazolium derivatives that have been recently studied to facilitate filling in the knowledge gaps.

## 1. Introduction

The term zeolite (from the Greek word “boiling stone”) was first coined by A. F. Crönstedt in 1756 [1]. These materials are widely used and still have much to offer [2]. New synthetic methodologies and new types of zeolites are published and patented each year [3,4,5]. Zeolites can be found in Nature, but they are also synthesized, which has been the focus of research in this area for years due to the large quantity of factors that affect the resulting products [6]. Among the parameters that influence the synthesis are organic structure-directing agents (OSDAs), which are organic molecules that have been shown to influence the type of zeolitic structure obtained. In particular, imidazolium-based cations are widely used and are still being studied to understand their effects as structure directors [7,8]. The focus of the present review is on imidazolium-based cations and the structures obtained when they are employed as OSDAs. The review is divided into two parts: a brief overview of zeolite and zeotype synthesis and a discussion on the structures obtained with imidazolium-based cations. Finally, some remarks and conclusions are presented.

## 2. Zeolites

Zeolitic materials are crystalline tectosilicates composed of TO_4_ tetrahedra linked together by the O atoms that are on the vertices [9,10]. Atoms represented by T can be tri- or tetravalent cations and create a wide range of possible zeolite compositions, from those made purely of silica to those with multiple combinations of Si and Al, passing through all possible isomorphic replacements with P, B, Ga, and Ge, and so on. Due to this fact and because zeolites are microporous and contain channels and cavities, cations are usually found in these cavities to compensate for the excess structural charge. Additionally, as indicated by the etymology of the term zeolite, there may be water molecules occluded in their pores. Then, the overall composition of zeolites can be described as follows:

M_x/n_[(T^III^O_2_)_x_(T^IV^O_2_)_y_]•zH_2_O

where n, x, y, and z are dependent on the synthetic method and the zeolite structure.

Zeolites can be found in Nature or synthesized in a laboratory. An important factor when studying the composition of zeolites, particularly the ones containing aluminum, is the avoidance of the formation of Al-O-Al bonds due to the energy cost of the resulting repulsions between two contiguous [AlO_4_] tetrahedra. This principle is known as the Loewenstein rule [11].

Currently, a criterion based on the framework density (FD, the number of T atoms per 1000 Å^3^) is used to distinguish zeolite and zeolite-like materials from denser tectosilicates [12]. The maximum FD for zeolites is 21 T atoms/1000 Å^3^; as an example, sodalite (SOD) has an FD of 16.7 T/1000 Å^3^.

### 2.1. Nomenclature and Classification

There are different ways of describing zeolites. The nomenclature accepted by the International Union of Pure and Applied Chemistry (IUPAC) is different from the formula introduced above and is as follows [13,14]:

ǀguest compositionǀ [host composition] _h_ {host structure} _p_ {pore structure} (Sym)–**IZA**

The term “IZA” is a three-letter code referring to the structural topology of the zeolite defined by the International Zeolite Association (IZA). Currently, there are 232 structural codes accepted (accessed on 07/22/2017) [12]. An example of this nomenclature is as follows:

ǀNa_12_ (H_2_O)_27_| [Al_12_Si_12_ O_48_] _h_{3 [4^6^]} _p_{0 [4^6^6^8^]/ 3 [4^12^6^8^8^6^] <100> (8-ring)}–**LTA**

Zeolites are mainly classified according to their microporosity [10] and channel system [15]. They can be divided into small pore zeolites, medium pore zeolites, large pore zeolites and extra-large pore zeolites (Table 1).

Zeolites can also be classified according to their channel system as 1-dimensional (e.g., AFI type), 2-dimensional (e.g., MOR) or 3-dimensional (e.g., MFI).

### 2.2. Construction

**Figure 1:**
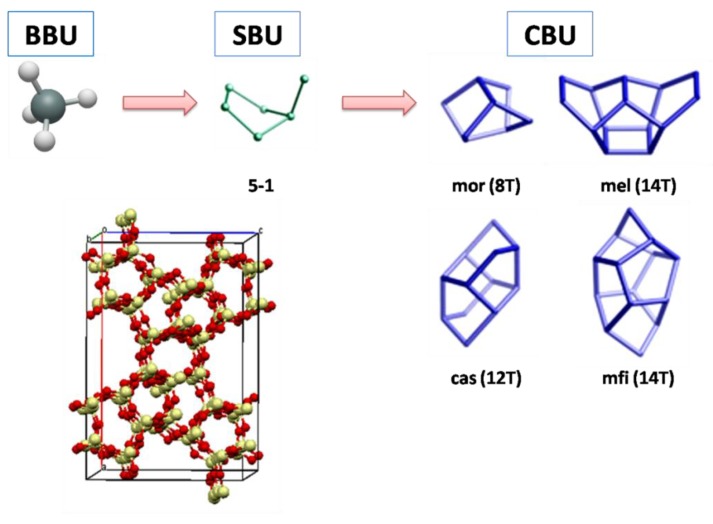
Construction of an MFI zeolite (adapted from the IZA Database website with permission from ref. 12, copyright © 2017 Structure Commission of the International Zeolite Association (IZA-SC) [12]).

All zeolites are based on the TO_4_ tetrahedron, which is designated the Basic Building Unit (BBU) [16]. The proper union of a small number of these groups up to 16 T atoms gives rise to the so-called Secondary Building Units (SBUs). Each SBU is designed from the assumption that the entire framework is composed of just one type of SBU. Composite Building Units (CBUs) are formed from a finite number of SBUs and reflect the characteristics of the zeolitic framework. Different types of CBUs can be found in a zeolitic framework, and their use helps to identify the relationship between zeolite types. The combination of SBUs and the CBUs usually describes the zeolitic framework. Figure 1 shows an example of the construction process for a better understanding; the union of BBUs forms SBUs (e.g., 5-1) and CBUs (e.g., mor, mel, cas and mfi), and their combination describes the zeolite (e.g., MFI).

**Figure 2:**
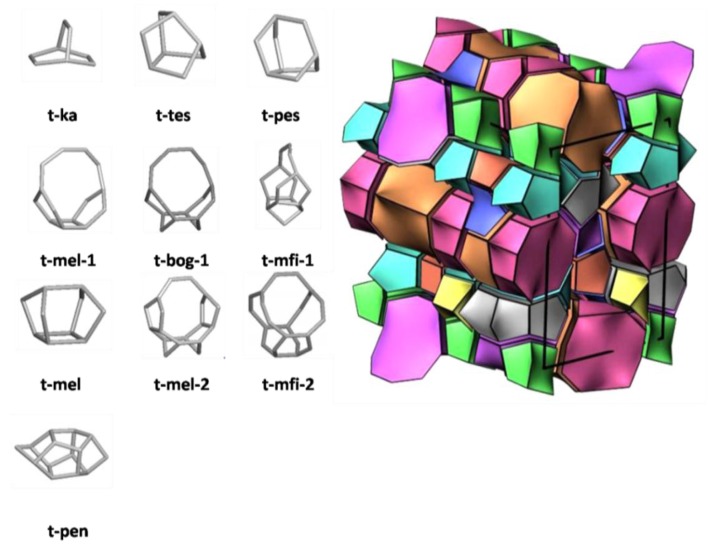
Construction of an MFI zeolite with natural tilings (adapted from the IZA Database website with permission from ref. 12, copyright © 2017 Structure Commission of the International Zeolite Association (IZA-SC) [12] and adapted from ref. 17, copyright © 2010 American Chemical Society [17]).

This arrangement has some ambiguities, which are solved with a new concept of natural tilings [17]. The tilings correspond to the cavities and cages or to the channel formations and are obtained by observing several rules described by Blatov et al. [18], such as: the tilings must maintain the symmetry of the framework, the faces of the tiles must be of similar sizes, and every face must be connected to others in the tiling (no face is by itself). Continuing with the example of the MFI zeolite, its natural tilings are found in Figure 2. Compared with the previous construction, some of the tilings are similar to the CBUs; for example, the CBU-mfi is identical to the natural tiling *t*-mfi-1, and the CBU-mel is equivalent to the natural tiling *t*-mel-1.

## 3. Zeotypes

The classical definition of zeolites included only the silicates and aluminosilicates [9]. Therefore, the introduction of phosphorous in the zeolitic framework was disruptive in chemical terms, as it opened a new field full of different materials that could be new or analogous to the aluminosilicates [19,20]. These materials were known as zeotypes, and some of them possessed interrupted open frameworks. The first substitution performed in the synthesis of these materials was to obtain an aluminophosphate or AlPO, which refers to the composition [21]. The introduction of silica into these materials gave the silicoaluminophosphates, or SAPOs. In the same way, it was possible to add other metals or elements to the framework of AlPOs or SAPOs, resulting in MeAPOs/ElAPOs or MeAPSOs/ElAPSOs.

As mentioned in the introduction, there are several *forbidden* bonds in zeolitic structures, so in these phosphorous-based materials, it was interesting to study the same property to deepen the knowledge of this type of material. For this reason, Flanigen et al. observed that several bonds seemed to be repeated and gave neutral or negatively charged frameworks, and other bonds were never observed, as they would have created positively charged frameworks [19]. These data are collected in Table 2.

As in aluminosilicates, Si-O-Si and Si-O-Al bonds were observed, but following the Loewenstein rule, Al-O-Al bonds were *forbidden* in zeotypes. Phosphorous acted similarly to aluminum in the sense that P-O-P bonds were also *forbidden*. In the case of aluminophosphates, Si atoms substituted for P atoms in the same way that Al substituted for Si in the pure silica frameworks, requiring one positively charged ion to compensate for the charge and imparting acidity on the resulting material. This substitution was the origin of the “silica islands” that will be explained later.

The differences between aluminophosphate and the typical aluminosilicate can be classified according to three main aspects: aluminum can coordinate 4, 5 or 6 oxygen atoms (5 coordination is related to interaction with fluoride anions), the strict alternation of the Al polyhedra and P tetrahedra gives an even number of T atoms (i.e., 8, 10, 12, 14, ...), and the zeotype framework and the organic structure-directing agents interact through van der Waals forces and hydrogen bonds [22]. The second aspect was already explained, and the third is going to be treated subsequently in this text. Focusing on the coordination of aluminum, in aluminosilicates, this element is mostly found in the tetrahedra in the framework and in an octahedral state when not incorporated [23]. However, there exist some exceptions, such as in the case of zeolite beta, which is synthesized in hydrofluoric media [24]. The aluminum was found to interact through oxygen with the fluoride anions. However, this octahedral state returned to tetrahedral by calcination of the sample, and sometimes, the tetrahedral state was also created by partial hydrolysis of the Al-O-Si bonds during the hydration process after calcination. In the case of AlPOs, 5- or 6-coordination was maintained but was not necessarily related to fluoride, but the interaction with fluoride also appeared in these cases [20,25].

**Figure 3:**
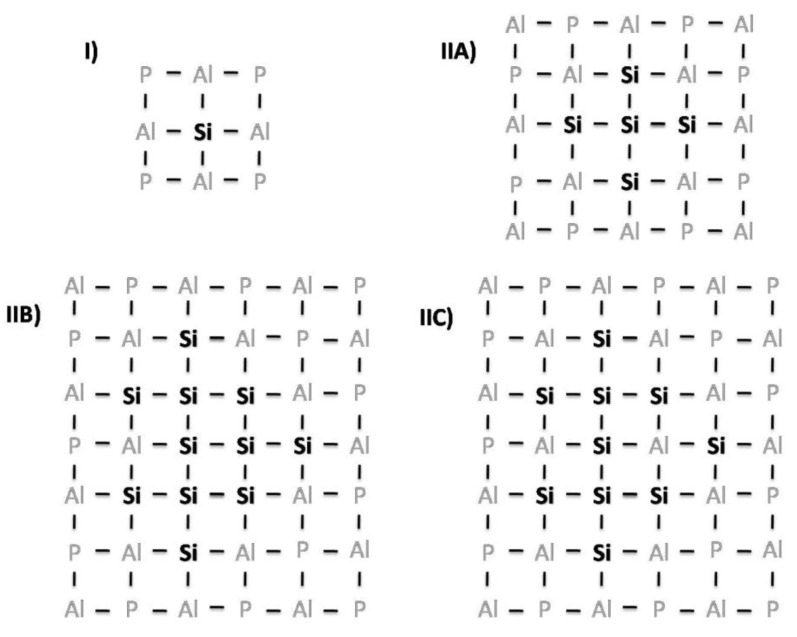
Distribution of Si in SAPOs: (**I**) isolated Si; (**II**) “silica islands” with 5 (IIA), 11 (IIB) and 10 (IIC) Si atoms (reprinted minimally modified from ref. 26 copyright © 1994 with permission from Elsevier [26]).

Barthomeuf proposed a topological model by which the introduction of silicon in the framework was defined, that is, if Si was isolated (Figure 3I) or directly bound to other Si tetrahedra, giving what was called “silica islands” [26]. This distribution is shown in Figure 3, omitting the oxygen bridges to facilitate its comprehension. There were three types of islands proposed at that time: 5-Si tetrahedra islands (Figure 3.IIA), 11-Si tetrahedra islands (Figure 3IIB) and 11-Si tetrahedra islands where one tetrahedron was replaced by an Al tetrahedron (Figure 3IIC). The importance of this distribution was reflected in the explanation of the different acidities of the SAPOs; for example, the acidity in presence of the “silica islands” was located on their edges. Several studies with respect to the presence of silicon islands in certain compositions of the samples were performed [27], resulting in the establishment of some compositional limits for certain zeotypes or in the recommendation of techniques that may help in the identification, such as ^29^Si MAS NMR [28].

Martens and Jacobs developed the isomorphic substitution mechanism (SM) by which they defined how the different atoms were incorporated in the framework [29]. The structures are classified as:

- SM I: substitution of the aluminum bound to phosphorous tetrahedra by monovalent (SM Ia), divalent (SM Ib) or trivalent (SM Ic) elements, resulting in M-O-P bonds.
- SM II: substitution of the phosphorous bound to aluminum tetrahedra by tetravalent (SM IIa) or pentavalent (SM IIb) elements, resulting in M-O-Al bonds.
- SM III: substitution of adjacent aluminum and phosphorous tetrahedra for two silicon tetrahedra.

## 4. Some Concepts on the Synthesis of Zeolites and Zeotypes

Some very interesting reviews summarize the history of zeolite and zeotype synthesis, such as the one by Masters and Maschmeyer [30] or the one published by Cundy and Cox [9]. In the present article, some concepts will be noted due to their importance and relation to imidazolium cations.

The first proven synthetic zeolite was a chabazite obtained by Barrer in 1948. To synthesize this zeolite, he mimicked the conditions under which these materials appeared in Nature, resulting in the well-known hydrothermal synthetic method. This method required high temperatures and autogenous pressure to obtain the desired materials. A higher pH and lower crystallization temperature were selected by Milton at the Linde laboratories in 1949 to solubilize the reagents.

In 1897, Ostwald observed that solids first crystallized in a less-stable crystal structure and then converted over time to a more-stable polymorph, which was called “Ostwald’s rule of stages” [31]. As a result of further observation, Ostwald combined this rule with the “Gibbs-Thomson effect”, resulting in what was known as “Ostwald ripening”, which stated that small crystals dissolved as larger crystals continued to grow. In the development of zeolite syntheses, these materials were also found to follow these rules, and they also included a series of intermediates that were less-stable than the final dense non-zeolitic phase [32]. These rules were essential for understanding the result of the scientists who started in zeolite synthesis. Currently, these rules are still important in the search for materials with chiral enantiopurity, such as those applied in the pharmaceutical industry [2].

In 1961, Barrer and Denny used tetraalkylammonium ion to obtain high-silica zeolites [30]. This was the moment when the concepts of templates and organic structure-directing agents began to be discussed. This discussion posed the question of the role of these compounds. Davis and Lobo proposed a classification system in which these compounds were divided into three groups: true templates, where the obtained zeolites adopted the geometric and electronic configuration of the “templating” molecule even after calcination; structure-directing agents, whose effect was to direct the formation of a specific type of zeolite; and space-filling compounds, which increased the thermodynamic stability of the organic framework composite [33].

In another review by Lobo, Zones and Davis, structure direction was discussed and inorganic cations and heteroatoms were also discussed as having an influence on the synthesis [34]. For example, certain zeolites could only be obtained in the presence of Na^+^ or K^+^. The authors summarized the important aspects of an organic structure-directing agent in comparison to what had been reported previously. Several of the directing agents were stable under the synthetic conditions, had certain rigidity, a moderate hydrophobicity and the ability to interact via van der Waals forces or hydrogen bonds. It was also discussed that the shape and radius of gyration influenced the type of zeolite synthesized and that it was important not to forget the distribution of charges.

From the high-silica zeolites, the next step was the search for pure silica zeolites, and in 1978, researchers from Union Carbide led by Flanigen published the synthesis of the first pure silica MFI zeolite, silicalite-1 [35]. The novelty of this synthesis was the start of the fluoride revolution, which had the same role as the hydroxide media (a mineralizer agent that helped to dissolve the reagents, including the silica) and was also involved in the subsequent concentration of the synthetic gels [36]. Since then, the use of HF became common in synthesis, being studied as frequently as the synthesis in basic media, not only to obtain pure silica zeolites but also zeolites with other heteroatoms incorporated in them, such as aluminum or germanium [37,38,39]. Some interesting aspects were the low number of defects, the location of the fluoride anions inside the D4R cages and the formation pentacoordinated Si, where one of the bonds was to the fluoride anion [40].

Flanigen and co-workers at Union Carbide were interested in the incorporation of new elements [30]. Therefore, in 1982, Wilson et al. published the first known study on aluminophosphates [21]. Since then, zeotypes have been studied under multiple conditions, such as in basic or hydrofluoric media [25,41].

As was mentioned, the use of fluoride media allowed for a decrease in the amount of water employed in the synthesis. In addition, from this point, several researchers started to think about solventless synthetic methods. One attempt to examine the possibility of not using water as the solvent in the synthesis was published by Althoff et al. in 1994, where the authors dried several reagents (including ammonium fluoride) at 650 °C and the precursors at 100 °C to ensure that there was no water in the initial mixture [42]. After different lengths of time in an autoclave at 180 °C, the authors found that an MFI phase was formed. Therefore, it was proposed that the silica reacted with ammonium fluoride through the following reaction:

$${\mathrm{SiO}}_{2}+4{\mathrm{NH}}_{4}F\to {\mathrm{SiF}}_{4}+4{\mathrm{NH}}_{3}+2H_{2}O$$

The authors argued that the amount of water created was not enough to reach the saturation pressure needed in this reaction, but they could not exclude the formation of a thin film of water on the surface or the presence of a small liquid phase constituted by capillary condensation. Nevertheless, a hypothesis based on vapor mass transfer with SiF_4_ as the mobile species was proposed. Some other factors had to be taken into account because the synthesis in these conditions was not and still is not simple or currently understand. Recently, Morris and James published a short article about this question [43]. The presence of very small amounts of water in what had been called water-free synthetic methods was identified, except in the case of solid-state reactions that were performed at extremely high temperatures.

Another trend in zeolite synthesis these last years has been the use of non-aqueous solvents, which meant that the main solvent was not water, as opposed to the solventless method established in the last paragraph. A review by Morris and Weigel in 1997 classified the organic solvents based on the strength of their interaction with silica through hydrogen bonding (high-medium, low-medium or non-hydrogen bonding), as these solvents could also act as templates, structure directors or space-filling compounds in the synthesis [44].

## 5. Imidazolium Ions in Synthesis of Zeolites and Zeotypes

Imidazolium-derivative cations are heterocyclic compounds without exocyclic conjugation that are obtained from the quaternization of the N in the corresponding imidazole derivative [45,46,47]. The resulting compounds are generally soluble in water, in contrast to the low solubility of the initial reagent, and have low melting and relatively high boiling points. These last characteristics and the fact that their vapor pressure is low are the reasons for considering the use of some of these organic salts as ionic liquids.

**Figure 4:**
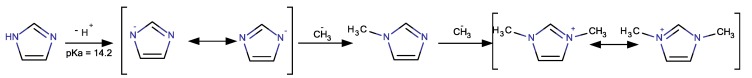
Synthetic path to 1,3-dimethylimidazolium (designed using the program Marvin Sketch [50]).

Different mechanisms for the synthesis of the cations have been proposed, which usually involve deprotonation of the imidazole derivative [48,49]. In Figure 4, one of the possible mechanisms for obtaining 1,3-dimethylimidazolium is shown:

The most stable tautomeric form resulting from the first methylation can be influenced by the type of substituent that the initial imidazole has at the C positions, which will decide the final product in the case of more complex imidazolium derivatives. As an example, the volume occupied by the substituents may indicate the preferred insertion position by enforcing substitution at the less hindered N.

A question that can arise is the importance of studying imidazolium derivatives and not limiting the studies to the quaternary ammoniums that have already been effective. First, it is important to note that the chemistry of the two types of compounds is different; imidazolium derivatives are heteroaromatic compounds that include the N atom in the aromatic ring [49,51]. The aromatic characteristic of the imidazolium derivatives helps to stabilize the positive charge. This aromatic ring also gives certain rigidity to the structure, as the ring is planar, allowing control of the flexibility through the substituents chosen for the study; meanwhile, quaternary ammonium derivatives are more flexible unless they are designed with planar substituents. Another characteristic of the imidazolium derivatives is the possibility of their use as solvents and structure-directing agents and their ability to be recycled [43,52]. Both characteristics will be addressed later. One last difference is that imidazolium derivatives and quaternary ammonium derivatives lead to different geometries and volumes, so both can enforce the synthesis of a wide variety of possible structures, which may be different between the two groups. Imidazolium cations and quaternary cations are not in competition; together their characteristics and possibilities may be used to better understand zeolites and to obtain different functional materials.

The first time that an ionic liquid (IL) was used in the synthesis of zeotypes was in 2004, and it was reported by Cooper et al. in the journal *Nature* [53]. The IL used, 1-ethyl-3-methylimidazolium bromide, led to the formation of the materials SIZ-1, SIZ-2, SIZ-3, SIZ-4, SIZ-5, SIZ-8 and SIZ-9. ILs are defined as compounds that melt at temperatures above 100 °C with low vapor pressure. However, when applied to the synthesis of zeolites, this temperature could be raised slightly to approximately 150–200 °C. This fact justifies their use as solvents. In that article, imidazolium derivatives were used as both solvents and templates, and the main characteristic was that they were fundamentally based on ions, despite the presence of very small quantities of water. At this point, the use of ionic liquids defined a new class of synthesis called “ionothermal synthesis” [54]. Since these compounds act as solvents and templates, the competition between the solvent-framework and template-framework interactions, which is a characteristic of the hydrothermal synthesis, is eliminated. It is important to remark that using a separate solvent from our OSDA could influence the structure obtained. Using the same molecule to fill a double role may help to study and better control the effects of directing groups; however, the other interactions could also result in products of interest. Therefore, neither type of synthesis is better to study, and both are needed.

ILs have also been used in hydrothermal syntheses where low quantities of water were added as a solvent and a form of hydroxide, and they have also been used in microwave syntheses. In the first case, ILs have facilitated the formation of new zeolites, such as HPM-1, or already-known zeolites with new morphologies, such as spherical ZSM-5 [55,56]. In the second case, these compounds were considered excellent microwave absorbents due to their high ionic conductivity and polarizability, and reviews have already been published on this topic [52,54]. In general, ILs have been used from room temperature to 200 °C with different compositions (e.g., pure silica, Si/Al, Si/Ge, AlPOs, and MAPOs) and in a variety of time periods (from hours to months).

Imidazolium derivatives are also related to a particular type of Metal-Organic Frameworks (MOFs) known as Zeolitic Imidazolate Frameworks (ZIFs) [57,58]. These materials are composed of imidazolate linkers and metal ions, and their structures resemble zeolites. These materials are not a subject of this review, but it is interesting to mention that ZIFs make up a whole new complete area, and several reviews have already been published for those who want to learn more about them [59,60].

Table 3 was constructed by reviewing the articles published using ILs in hydrothermal or ionothermal syntheses. Due to the complexity and enormous variability in the synthetic conditions, the imidazolium-based cation name, the types of frameworks that were obtained (pure phases, such as pure silica, aluminosilicate, or other zeotypes), and some recent references for each are presented, which may help researchers with their projects. The new types of zeolites and the materials whose structures are still not classified into the three-letter zeolite types have been marked with a symbol.

As was explained at the beginning of this section, ionic liquids are characterized by having a low melting point and relatively high boiling point [54,55,56]. Therefore, it can be assumed that their decomposition temperatures will also be high. However, it is still necessary to confirm that the OSDAs do not decompose during synthesis. For this reason, it is recommended to use techniques such as CHN elemental analysis, ^1^H- and ^13^C-MAS NMR (magic angle spinning nuclear magnetic resonance) spectroscopy, and/or infrared spectroscopy to help identify the imidazolium ring and its substituents and other possible contaminants [51,61,62]. The table below contains examples where the imidazolium derivative was not decomposed as far as we know.

By observing the zeolite type obtained in each case, it was possible to identify several relationships with the role of the OSDA. First, the imidazolium derivatives act as space-fillers, which means that the volume and geometry of the compounds define the obtained zeolites. Generally, larger imidazolium derivatives should lead to the synthesis of zeolites with larger cavities. Several examples of this property can be seen in the table. The MFI-type zeolite seemed to be associated with imidazolium derivatives that have terminal alkyl chains of more than two atoms, such as 1-butyl-3-methylimidazolium, or bridging alkyl chains, such as 1-(propan-2-yl)-3-(10-[1-(propan-2-yl)-1*H*-imidazol-3-ium-3-yl]decyl)-1*H*-imidazol-3-ium. TON zeolites were often synthesized from the same imidazolium derivatives as the MFI zeolite, such as with 1,3-diethylimidazolium or 1-(propan-2-yl)-3-(6-[1-(propan-2-yl)-1*H*-imidazol-3-ium-3-yl] hexyl)-1*H*-imidazol-3-ium. Therefore, it may be possible to obtain the MFI type in cases where the TON zeolite was already achieved and vice versa. The length of the terminal alkyl chains has also been an object of study, such as in the article by Wen et al. [87]. The authors studied alkyl chains with lengths from two to 14 carbon atoms and concluded that the lower numbers of carbon atoms (two and four) formed pure TON phases, an intermediate number of carbon atoms (six) gave mixtures of MFI and TON zeolites, and longer alkyl chains (>eight) resulted in magadiite and/or amorphous phases. Therefore, it was concluded that there are lower and upper limits to the number of carbons in the terminal alkyl chains that can direct the formation of MFI or TON zeolites.

Another two types of zeolites seemed to follow a similar rule: BEA* and FAU. Both have been synthesized using imidazolium derivatives with voluminous lateral groups, such as 1,3-diisobutyl-imidazolium or 1,3-bis(cyclohexyl)imidazolium. Comparing the cell parameters or framework densities, the two zeolite types appear to be very different at first sight [12]. However, the volume of the molecules that can diffuse through them (5.95 Å for BEA* and 7.35 Å for FAU) are similar; therefore, this may be the key to the same imidazolium derivatives forming these two types of zeolites. In the case of the RTH and STW types, which also share imidazolium derivatives, such as 2-ethyl-1,3,4-trimethylimidazolium, their framework densities are similar (16.1 T/1000 Å^3^ for RTH and 16.4 T/1000 Å^3^ for STW), as are the molecules that can diffuse through them (from 1.67 to 4.14 Å for RTH and from 3.38 to 4.88 Å for STW) [12]. In addition, a final example of these relationships is the MTW type zeolite, which has been obtained on several occasions from imidazolium derivatives with bridging alkyl chains, such as 1-(propan-2-yl)-3-(14-[1-(propan-2-yl)-1*H*-imidazol-3-ium-3-yl] tetradecyl)-1*H*-imidazol-3-ium.

It is also important to note that imidazolium derivatives have certain control of the flexibility around the imidazolium ring; that is, the substituent can be chosen based on their conformational possibilities. An example of this fact is the work published by Variani et al. [84]. The cations 1,2,3-triethylimidazolium (123TEI) and 1,2,3-triethyl-4-methylimidazolium (123TE4MI) contain several rotatable bonds, leading to the formation of three and four most-stable non-equivalent conformers, respectively. Based on the corresponding computational study, it was explained that 123TEI fits nicely inside the MFI zeolite and that, initially, this zeolitic structure was not expected with 123TE4MI. Therefore, to a certain extent, it is possible to predict and control the zeolitic structures that will be obtained by designing the substituents and performing conformational analysis of the OSDAs.

Second, the imidazolium derivatives can also exhibit a templating effect in which their directing influence is not only established by their geometry but also by their electronic configuration. Imidazolium cations are able to form π-π interactions with each other through their imidazolium rings [7], which may help to organize the BBUs and influence the final product of a synthetic method. Even though this property has not yet been studied with these cations, some interesting studies along this line have already been published, such as the self-assembly of ephedrine and pseudoephedrine reported by Bernardo-Maestro et al. [88]. The computational analysis of the resulting zeolites has been successful in explaining the zeolite types in thermodynamic terms by calculating the energies of the most stable conformations and the locations of the imidazolium derivatives in the framework [65,84]. The host-guest relationships are mainly due to the electronic terms, and studies such as those of Rojas et al. and Variani et al. demonstrated how the cation-framework was organized in the cases of the MFI, MTW and STW zeolites.

The last role of OSDAs that was described in Section 4 is the role of pure structure-directing in which a molecule forms only one type of zeolite. However, this role should be adapted to each condition; it is not the same to synthesize a pure silica zeolite than a silicoaluminophosphate. Not taking this into account may just lead to an imidazolium that fulfils this pre-requisite, namely, 1,1′,1′′-(2,4,6-trimethylbenzene-1,3,5-triyl)tris(methylene)tris-(3-methyl-1*H*-imidazol-3-ium), but further studies and tests of different conditions would probably lead to other zeolite types. Therefore, in this sense, it is interesting to continue studying different conditions with different imidazolium derivatives because to date, there is not enough proof to state that any imidazolium derivative is a pure structure-directing agent for only one zeolite type in the presence of different heteroatoms. In this sense, studying the hydrophobicity of the OSDAs can help explaining their role. Kubota et al. [89] already studied this parameter in ammonium derivatives by partitioning the organic cations between water and chloroform. The host/guest thermodynamic stability of the material is enhanced by the increased stability of the components in the initial mixture. The authors concluded that the best ammonium derivatives for pure silica zeolite synthesis were the rigid, moderately hydrophobic, bulky molecules with the longest axis below 10 Å. The influence of hydrophobicity has begun to be studied in the case of imidazolium derivatives, as reported by Rojas et al. [65]. The derivative 1-benzyl-3-methylimidazolium (1B3MI) showed a slightly greater hydrophobicity character than 1-benzyl-2,3-dimethylimidazolium (1B23DMI), and for this reason, it was predicted to perform better as an SDA.

Finally, the continued study of imidazolium derivatives in zeolite synthesis is important, as they proved to be interesting in their ability to form new types of zeolites [3,25,66] for new applications and revealed a new synthetic methodology [53], as discussed earlier. The use of imidazolium derivatives is also considered a green chemistry process because it is possible to recover and reuse them [66]. As observed in the table of derivatives, there are still many imidazoliums and possible conditions remaining to be researched. Determining the relationships between imidazolium derivatives and the zeolite types will lead to a better understanding of the synthesis and the ability to control the final products.

## 6. Conclusions

In this article, the state of the art techniques relevant to the synthesis of zeolites with imidazolium derivatives were summarized, and a brief introduction on zeolites and zeotypes was presented. Several questions related to the synthesis and structure of these materials were addressed based on analysis of the literature. It was shown that imidazolium derivatives can act as space-fillers, templates and structure-directing agents. Finally, a list of ionic liquids employed in zeolite and zeotype syntheses was presented in relation to the obtained zeolitic framework in an attempt to help researchers with experimental planning and with the discussion of results in an area that it is important for zeolitic research and is difficult to keep up to date.

## Figures and Tables

**Table 1:** Classification of zeolites according to their pore size.

Pore Size	Number of Tetrahedra (MR ^1^)	Pore Diameter (Å)	Example
Small	8	4	PST-1 (NAT)
Medium	10	5.5	ZSM-5 (MFI)
Large	12	7.5	ZSM-12 (MTW)
Extra-large	>12	>7.5	CIT-5 (CFI)

^1^ MR: Members of the ring.

**Table 2:** Bonds observed in zeotypes and bonds not possible in zeotypes ^1^.

Observed Bonds	Impossible Bonds
Al-O-P	P-O-P
Si-O-Si	P-O-Si
Si-O-Al	Al-O-Al
M-O-P	M-O-Al
M-O-P-O-M	M-O-M

^1^ M stands for other elements.

**Table 3:** Imidazolium-based cations used in zeolite and zeotype syntheses (the cations were designed with the program Marvin Sketch [50]).

Cation		Type
Neutral compound		
Imidazole	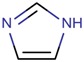	[ImidH]AlP_2_O_8_H_2_·2H_2_O, [ImidH]Al_3_P_4_O_16_H [63]
Bi-substituted imidazolium		
1-Benzyl-3-methylimidazolium	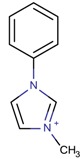	AFI, LTA [64], MFI, MTW [65]
3-Methyl-1-[(2-methylphenyl) methyl]-1*H*-imidazol-3-ium	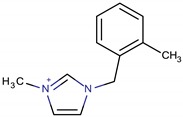	NUD-2 ^¥^ [66]
3-Methyl-1-[(3-methylphenyl) methyl]-1*H*-imidazol-3-ium	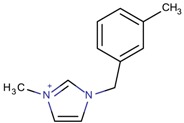	CIT-13 ^¥^, MFI [3], NUD-2 ^¥^ [66]
1-[(3,5-Dimethylphenyl)methyl]-3-methyl-1*H*-imidazol-3-ium	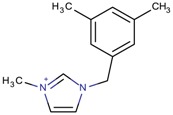	CIT-13 ^¥^ [3]
1,3-Bis(1-adamantyl) imidazolium	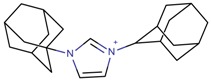	AFX, CFI, FAU, MOR, STF [67]
1,3-Bis(bicyclo[2.2.1]heptan-2-yl)imidazolium	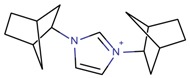	BEA*, SSZ-70 ^¥^ [67]
1,3-Bis(cycloheptyl) imidazolium	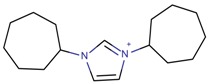	BEA*, MOR, SSZ-70 ^¥^ [67]
1,3-Bis(cyclohexyl)imidazolium	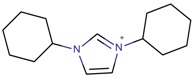	BEA*, EUO, FAU, MOR, SSZ-70 ^¥^ [67]
1,3-Bis(cyclohexylmethyl) imidazolium	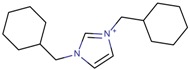	BEA*, FAU, MOR, MTW, SSZ-70 ^¥^ [67]
1,3-Bis(cyclopentyl) imidazolium	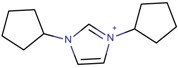	BEA*, SSZ-70 ^¥^ [67]
1,3-Bis(cyclooctyl)imidazolium	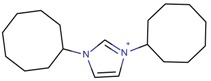	BEA*, FAU, MOR [67]
1,3-Bis(pentan-3-yl)imidazolium	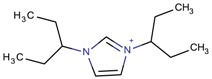	FAU, MTW, MOR, SSZ-70 ^¥^ [67]
1,3-Bis(*tert*-butyl)imidazolium	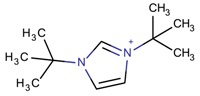	FAU, MOR [67]
1,3-Bis(2,4,4-trimethylpentan-2-yl)imidazolium	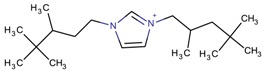	FAU [67]
1-Butyl-3-methylimidazolium	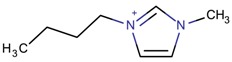	AEL [68], AFI [64], ANA, BEA*, MFI [8,56,69], FAU [70], LTA [71], TON [8,72], UWY ^$^ [73]
1,3-Dimethylimidazolium	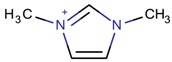	ITW ^$^ [74], FER [75], TON [67]
1,3-Diethylimidazolium	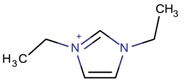	MFI, TON [67]
1,3-Diisopropylimidazolium	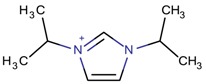	MTT, MTW, SSZ-70 ^¥^ [67]
1,3-Diisobutylimidazolium	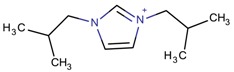	BEA*, FAU, MOR, MTW, SSZ-70 ^¥^ [67]
1-Ethyl-3-methylimidazolium	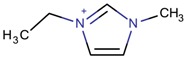	AEI, SIV ^$^, SOD [76], AEL, CHA, LTA [64], CLO [71], TON [77], UOS ^$^ [78]
1-Hexyl-3-methylimidazolium	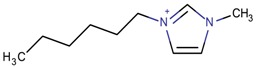	LTA [71]
1-Isopropyl-3-methyl imidazolium	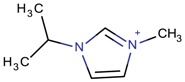	MRE*, MTT [79]
1-Methyl-3-pentylimidazolium	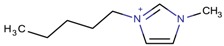	LTA [71]
1-Methyl-3-propylimidazolium	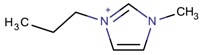	LTA [71]
1-(Propan-2-yl)-3-(4-[1-(propan-2-yl)-1*H*-imidazol-3-ium-3-yl]butyl)-1*H*-imidazol-3-ium	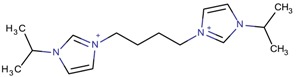	MTW, TON [79]
1-(Propan-2-yl)-3-(5-[1-(propan-2-yl)-1*H*-imidazol-3-ium-3-yl] pentyl)-1*H*-imidazol-3-ium	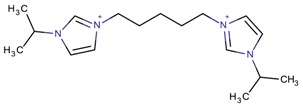	EUO, MFI, MRE* [79]
1-(Propan-2-yl)-3-(6-[1-(propan-2-yl)-1*H*-imidazol-3-ium-3-yl] hexyl)-1*H*-imidazol-3-ium	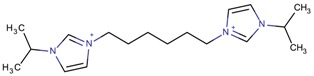	EUO, MFI, MRE*, MTW, TON [79]
1-(Propan-2-yl)-3-(7-[1-(propan-2-yl)-1*H*-imidazol-3-ium-3-yl] heptyl)-1*H*-imidazol-3-ium	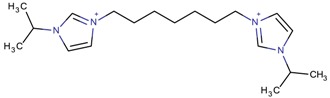	MTT [79]
1-(Propan-2-yl)-3-(8-[1-(propan-2-yl)-1*H*-imidazol-3-ium-3-yl] octyl)-1*H*-imidazol-3-ium	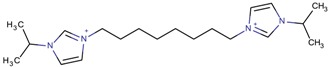	MRE*, MTT, MTW [79]
1-(Propan-2-yl)-3-(9-[1-(propan-2-yl)-1*H*-imidazol-3-ium-3-yl] nonyl)-1*H*-imidazol-3-ium	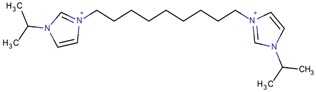	MFI, MRE*, MTW [79]
1-(Propan-2-yl)-3-(10-[1-(propan-2-yl)-1*H*-imidazol-3-ium-3-yl] decyl)-1*H*-imidazol-3-ium	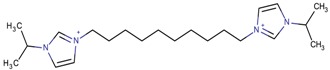	MFI, MRE*, MTW, NES, TON [79]
1-(Propan-2-yl)-3-(11-[1-(propan-2-yl)-1*H*-imidazol-3-ium-3-yl] undecyl)-1*H*-imidazol-3-ium	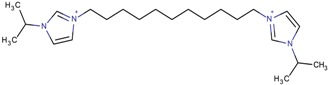	MTT [79]
1-(Propan-2-yl)-3-(12-[1-(propan-2-yl)-1*H*-imidazol-3-ium-3-yl] dodecyl)-1*H*-imidazol-3-ium	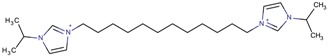	MRE*, MTT [79]
1-(Propan-2-yl)-3-(14-[1-(propan-2-yl)-1*H*-imidazol-3-ium-3-yl] tetradecyl)-1*H*-imidazol-3-ium	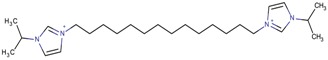	FER, MTW [79]
1-(Propan-2-yl)-3-(16-[1-(propan-2-yl)-1*H*-imidazol-3-ium-3-yl] hexadecyl)-1*H*-imidazol-3-ium	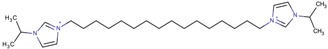	FER [79]
1-(Triethoxysilyl propyl)-3-dodecylimidazolium	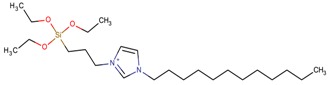	MFI [80]
1-(Triethoxysilyl propyl)-3-methylimidazolium	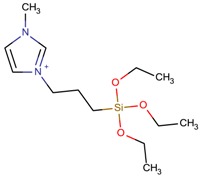	MFI [80]
Tri-substituted imidazolium		
1-Benzyl-2,3-dimethyl imidazolium	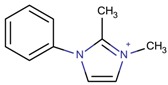	ITW ^$^, MTW [65]
2,3-Dimethyl-1-[(2-methyl phenyl)methyl]-1*H*-imidazol-3-ium	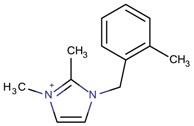	CIT-13 ^¥^, IWS ^$^, LTA [81]
2,3-Dimethyl-1-[(3-methyl phenyl)methyl]-1*H*-imidazol-3-ium	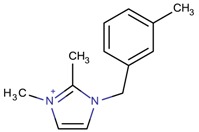	CIT-13 ^¥^ [3]
2,3-Dimethyl-1-[(4-methyl phenyl)methyl]-1*H*-imidazol-3-ium	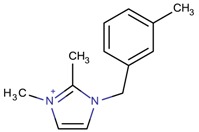	BEC, LTA [81]
1-[(3,5-Dimethylphenyl) methyl]-2,3-dimethyl-1*H*-imidazol-3-ium	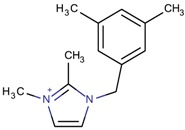	CIT-13 ^¥^ [3]
1-((2-[(1,2-Dimethyl-1*H*-imidazol-3-ium-3-yl)methyl]phenyl)-methyl)-2,3-dimethyl-1*H*-imidazol-3-ium	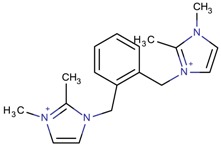	BEA* [81]
1-((3-[(1,2-Dimethyl-1*H*-imidazol-3-ium-3-yl)methyl]phenyl)-methyl)-2,3-dimethyl-1*H*-imidazol-3-ium	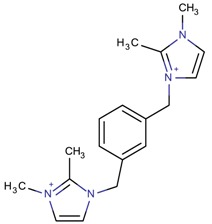	BEA*, IWS ^$^ [81]
1-((4-[(1,2-Dimethyl-1*H*-imidazol-3-ium-3-yl)methyl]phenyl)methyl)-2,3-dimethyl-1*H*-imidazol-3-ium	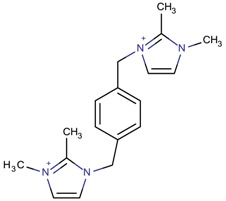	BEA*, BEC [81]
1-(2,3-Dihydroxipropyl)-2,3-dimethylimidazolium	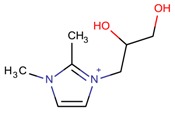	AFI [82]
2-Ethyl-1,3-dimethyl imidazolium	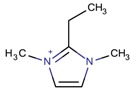	CSV ^$^, ITW ^$^, MTW, RTH, STF, STW^$^ [75]
1-Ethyl-2,3-dimethylimidazolium	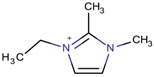	AFI [83], ITW ^$^, MTW [32]
2-Isopropyl-1,3-dimethylimidazolium	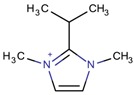	MOR, STF [75]
1,1′,1′′-(2,4,6-Trimethyl benzene-1,3,5-triyl)tris (methylene)tris-(3-methyl-1*H*-imidazol-3-ium)	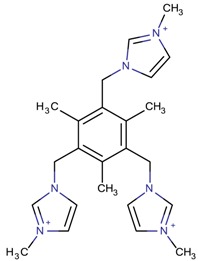	-ITV ^$^ [4]
1,2,3-Trimethylimidazolium	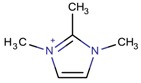	AFI, CHA, HPM-3 ^¥^ [25], ITW ^$^ [78], RTH [75], MFI, STF [84]
1,3,4-Trimethylimidazolium	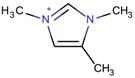	ITW ^$^, TON [74], MOR, RTH [75]
1,3,5-Tris(1,2-dimethyl imidazolium)benzene	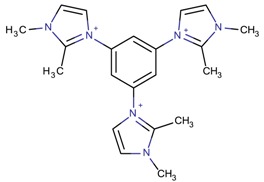	IWR ^$^, LTA [85]
Tetra-substituted imidazolium		
2-Ethyl-1,3,4-trimethyl imidazolium	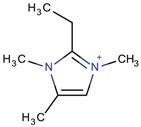	ITW ^$^, MTF, STW ^$^ [5,55], MOR, MTW, RTH [75]
1,2,3,4-Tetramethyl imidazolium	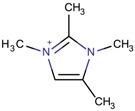	MOR, RTH, STW ^$^ [75]
1,2,3-Triethyl-4-methylimidazolium	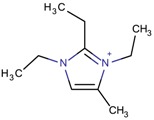	STF [84]
Penta-substituted imidazolium		
1,2,4,5-Tetramethyl-3-[3-(tetramethyl-1*H*-imidazol-3-ium-3-yl)propyl]-1*H*-imidazol-3-ium	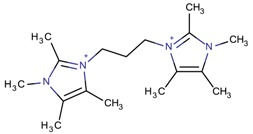	CSV ^$^, IWV ^$^, STW ^$^ [86]
1,2,3,4,5-Pentamethyl imidazolium	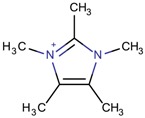	RTH, STW ^$^ [75]

^$^ New zeolitic framework type. ^¥^ Materials in study.
